# Notes

**Published:** 1896-08

**Authors:** 


### Notes.

The third International Congress of Dermatology will meet in London, from August 4th to 8th, of this year. The general programme has been published in a recent number of this journal.

There will be a museum of drawings, casts, models, naked eye preparations, microscopic specimens, works and atlases pertaining to diseases of the skin. There will also be an exhibition of clinical cases and demonstrations of the same, at 9 A. M. and 2 P. M. of August 5th, 6th and 7th, and at 9 A. M. of August 8th. Any one having anything to contribute to this department, will please address Dr. Jas. Galloway, 21 Queen Anne Street, Cavendish Square, W.

There will be an exhibition of cultures and microscopical preparations of organisms connected with the skin and its diseases. Any communications in regard to this department should be addressed to H. G. Plimmer, Esq., Wunderbau, Sydenham, London.

The social side of the Congress will be: 1st, an informal reception at the International Hall, Piccadilly Circus, on August 3d, from 9 to 12 P. M. 2d, a reception by the Lord Mayor and Lady Mayoress, at the Mansion House, on August 5th, from 9 to 11 P. M. 3d, a dinner to the foreign members, at the Hotel Cecil, on August 7th.

It is advised that foreigners should arrive in London not later than Sunday, August 2d, as Monday, the 3d inst., is a public holiday. Information in regard to hotels will be furnished on application to George Pernet, Esq., 77 Upper Gloucester Place, London, N. W.

GEORGE THOMAS JACKSON, M. D.
Foreign Secretary for the U. S.

---

The American Microscopical Society will hold its nineteenth annual meeting in the new Carnegie Library Building, Pittsburg, Pa., Tuesday, Wednesday, Thursday, and Friday, August 18, 19, 20, and 21, 1896. A hearty welcome will be extended to all interested in the microscopical sciences. Applications for membership and titles of papers to be read at the meeting should be addressed to A. Clifford Mercer, M. D., President, Syracuse, N. Y., or to Wm. C. Krauss, M. D., Secretary, 382 Virginia street, Buffalo, N. Y.

